# Metastatic lobular carcinoma of the breast found incidentally on pathology following cholecystectomy for chronic cholecystitis: A case report

**DOI:** 10.1016/j.ijscr.2021.01.106

**Published:** 2021-02-04

**Authors:** Alona Salita, Marcos Rosado, Kita Mack, John Pui, Richard Zekman, Kelly Dinnan

**Affiliations:** Beaumont, 28050 Grand River Avenue, Farmington Hills, MI, 48336, United States

**Keywords:** Breast cancer, Gallbladder metastases, Cholecystitis, Cholecystectomy, Case report

## Abstract

**Background:**

Traditional oncologic pattern of spread of breast cancer is metastasis to axillary lymph nodes, lung, liver and bone (Doval et al., 2006 [1]). Here we present a case of unknown synchronous breast cancer in a patient that was revealed on histopathologic assessment following elective cholecystectomy.

**Case summary:**

A 57 year old female presented for an elective laparoscopic cholecystectomy secondary to biliary colic. Histopathologic assessment of the gallbladder revealed metastatic adenocarcinoma with signet ring features, consistent with metastatic lobular carcinoma. The patient went on to have a complete oncologic workup that revealed invasive ductal carcinoma with components of high grade ductal carcinoma in situ in the left breast, lobular carcinoma in the right breast, and metastatic lobular carcinoma to left and right axillary lymph nodes as well as diffuse osseous metastatic disease.

**Conclusions:**

Metastatic disease to the gallbladder found incidentally on elective cholecystectomy is a rare presentation of synchronous breast cancer.

## Highlights

• Breast cancer rarely metastasizes to the gallbladder.
• Invasive lobular carcinoma with gallbladder metastases.
• Gallbladder metastases from the breast identified after elective cholecystectomy for chronic cholecystitis.
• Bilateral breast cancer with invasive lobular carcinoma in one breast, invasive ductal carcinoma in contralateral breast.
• Treatment of metastatic breast cancer with neoadjuvant chemotherapy.

## 1 Introduction

The second most common type of invasive breast cancer is invasive lobular carcinoma (ILC). ILC usually presents as a mass in the breast, either via breast exam or screening mammography, and diagnosed on biopsy. The mean age of presentation is 57 years old and there are several risk factors which include hormone therapy, age at menarche and age of first birth [2]. Breast cancer metastasis to the gallbladder is rare. Usual metastatic sites include lung, liver and bone [3]. Traditional oncologic pattern of spread of breast cancer is metastasis to axillary lymph nodes, lung, liver and bone [1,3].

Here we present a rare case of metastatic lobular breast carcinoma presenting incidentally on histopathology after cholecystectomy for biliary colic as the first clinical presentation of synchronous breast cancer.

## 2 Case presentation

**Fig. 1:**
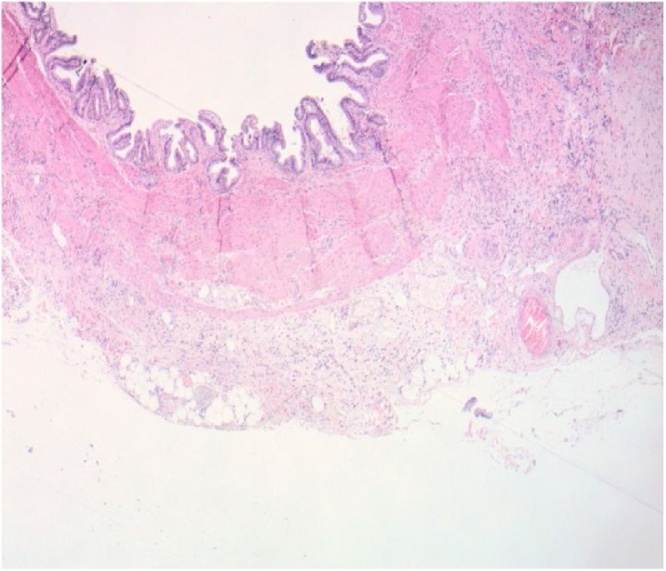
A low power view of the gallbladder from the cholecystectomy shows a moderately dense infiltrate of mononuclear cells in the muscularis propia and pericholecystic subserosal tissue (H & E, 40× original magnification) Fig. 1. A low power view of the gallbladder from the cholecystectomy shows a moderately dense infiltrate of mononuclear cells in the muscularis propria and pericholecystic sub-serosal tissue (H & E, 40× original magnification).

**Fig. 2:**
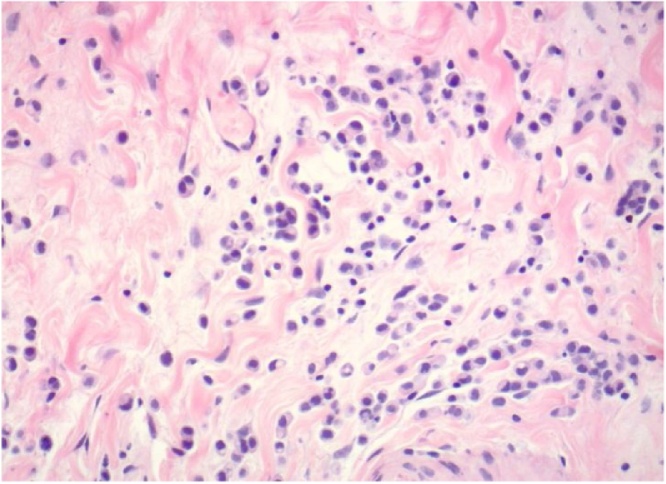
On higher magnification, the majority of the mononuclear cells have an eccentrically placed nucleus with many showing a perinuclear vacuole (H & E, 200× original magnification).

**Fig. 3:**
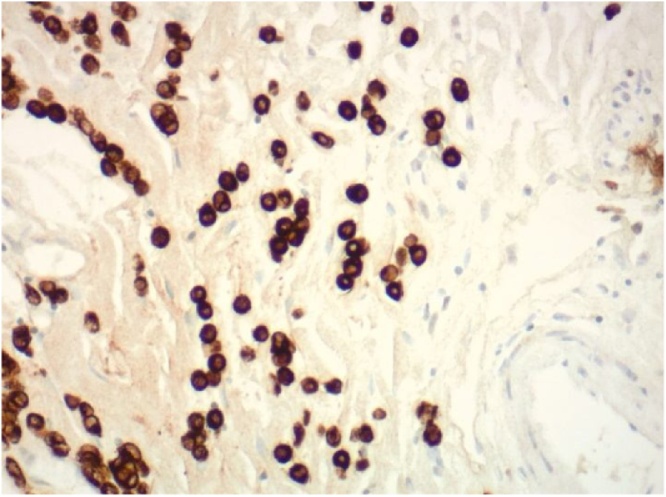
Immunohistochemical stains reveal that the mononuclear cells are positive for pancytokeratin, consistent with metastatic carcinoma (CK 88, 200× original magnification). Additional immunohistochemical stains reveal these lesional cells are positive for CK 7, GCFDP-15, and GATA3, and negative for CK 20 and E-cadherin. This profile is consistent with metastatic mammary lobular carcinoma.

This is the case of a 57 year old female who presented to the surgical clinic with a one month history of attacks of right upper quadrant abdominal pain, nausea and vomiting. An abdominal ultrasound demonstrated the presence of cholelithiasis and biliary sludge. The patient was diagnosed with symptomatic cholelithiasis and laparoscopic cholecystectomy was recommended to the patient on an elective basis. At the beginning of the procedure the peritoneal cavity was inspected with no masses, scar tissue or other abnormal pathology noted. During dissection of the gallbladder there were chronic omental adhesions noted but the gallbladder was not acutely inflamed. The cholecystectomy was able to be performed without complication and the gallbladder was sent for histopathological assessment. The pathology report was consistent with metastatic adenocarcinoma with signet ring features consistent with metastatic lobular carcinoma as well as chronic cholecystitis and cholelithiasis (see Fig. 1, Fig. 2). Breast biomarker studies performed on the gallbladder were significant for PASD positive staining, antibodies that were positive included CK88, CK7, BRST2, and GATA3 (see Fig. 3). Estrogen and progesterone receptors were both strongly positive, HER2 was negative.

At her follow-up appointment in the office a focused breast history and physical exam was performed. The patient denied family history of breast cancer. Full risk stratification and oncologic workup was pursued. Her BMI was 32.4, age of menarche was 13, her first pregnancy was at age 35, menopause began at 37 years old at which time she briefly was placed on estrogen therapy. She denied family history of cancer. She had a right breast lumpectomy in 2012 with benign pathology. Her last mammogram was in 2018 with no concerning findings at that time. A Gail score was calculated revealing a 12.5 % lifetime risk of breast cancer.

**Fig. 4:**
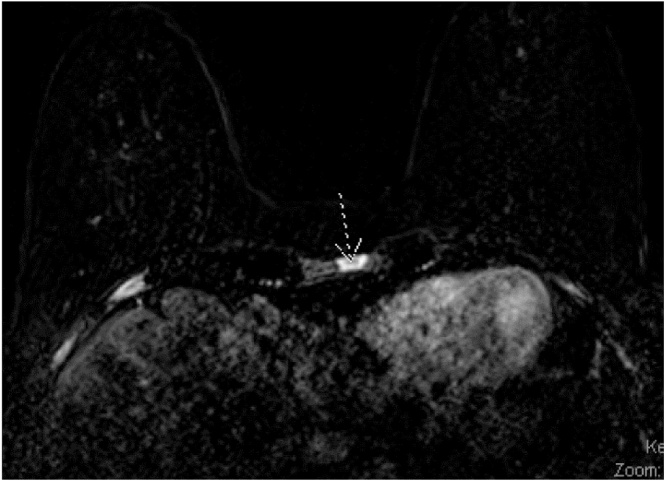
Sternal osseous metastasis suspicious masses on breast MRI.

Breast MRI revealed several spiculated masses in her right and left breasts as well as suspicious bilateral axillary lymph nodes. Potential areas of osseous metastasis were noted in her sternum and several ribs (see Fig. 4). The study was deemed as BI-RADS 5 and she was sent for ultrasound guided biopsy.

Ultrasound guided biopsies of these masses revealed:

i) Left breast: 11:00 spiculated mass, invasive ductal carcinoma with components of high grade ductal carcinoma in situ
ii) Left axillary lymph node: metastatic lobular carcinoma
iii) Right breast: 9:00 11 cm from nipple, lobular carcinoma
iv) Right axillary lymph node: metastatic lobular carcinoma

**Fig. 5:**
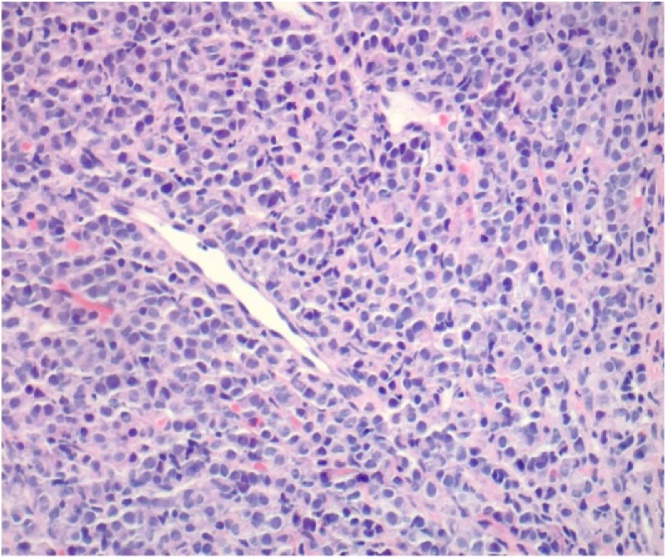
Subsequent radiographically guided core biopsies of left and right axillary and right intramammary lymph nodes show metastatic mammary lobular carcinoma with the same histologic and immunohistochemical appearance as that in the gallbladder (left axillary lymph node, H & E, 200× original magnification).

**Fig. 6:**
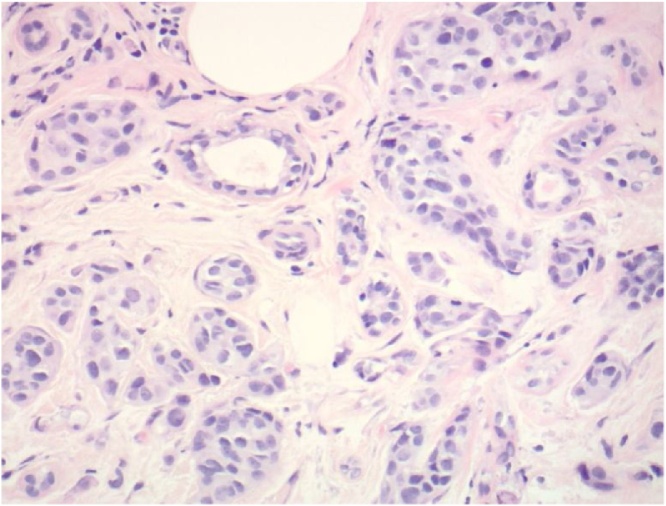
A radiographically guided core biopsy of a spiculated mass in the left breast shows intermediate grade invasive mammary ductal carcinoma (left breast, H & E, 200× original magnification).

All locations showed immunohistochemistry that consisted of estrogen receptor positive, progesterone receptor positive, and HER 2 negative. The samples were CK88 positive; CD3, CD20 and CD21 were all normal (see Fig. 5, Fig. 6).

**Fig. 7:**
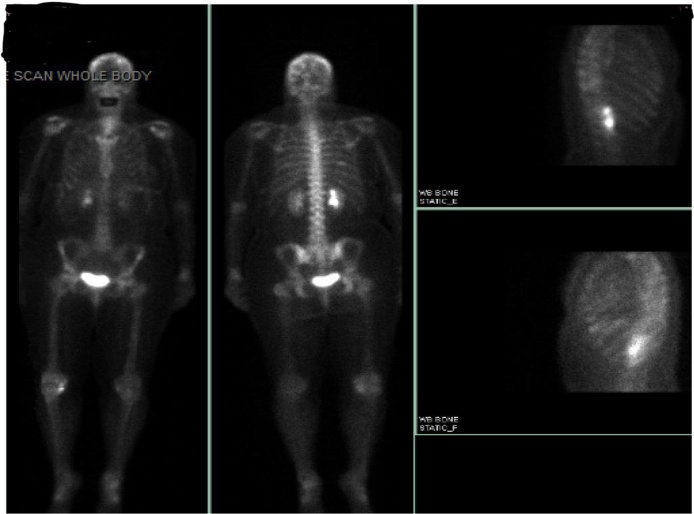
Whole body scan showing osseous metastasis to calvarium and posterior 9th rib.

Whole body bone scan demonstrated multiple foci of increased radiotracer activity within the calvarium and posterior ninth rib (see Fig. 7). This imaging grossly under detected the extent of osseous metastases that was detected on MRI of her brain which showed significant diffuse bony metastasis including the calvaria. There were no metastatic brain lesions noted.

The patient was initially treated with a CDK4/6 inhibitor (Palbociclib) which is a category 1 preferred first line treatment option for hormone receptor positive, HER2 negative metastatic breast cancer per the NCCN guidelines. She was also treated with zoledronic acid to reduce skeletal related events given the extensive osseous metastases as well as letrozole given her hormone receptor status. There are plans for restaging every 3 months.

## 3 Discussion

Breast cancer is the most common malignancy in females. According to the American Cancer Society’s Cancer Facts and Figures for 2020, breast cancer has the highest predicted new cancer cases for the year with an estimated 276,480 cases [4]. Due to advances in mammography and screening protocols for breast cancer it is being diagnosed and treated earlier with a 91 % total relative 5 year survival rate and a 99 % 5 year survival rate for early stage disease. The most common histologic subtypes are invasive ductal carcinoma (75–80 %) and invasive lobular carcinoma (10–15 %). The most common sites of metastases in breast cancer are brain, bone, lung, and liver. Various studies have demonstrated that there are different metastatic patterns depending on the histologic subtype of breast cancer. In a 2019 study by Mathew et al., they used a prospective collated database to analyze 960 patients with metastatic breast cancer in order to determine the metastatic patterns of invasive lobular carcinoma. They found that the most common sites of metastases in invasive lobular carcinoma was to the bone and GI tract while the most common sites of metastases in invasive ductal carcinoma were the lung and/or pleura and liver [5]. Invasive lobular carcinoma has also been found to be metastatic to gynecologic and retroperitoneal structures [4].

Laparoscopic cholecystectomy is one of the most common surgical procedures in the US with approximately 300,000 cholecystectomies being performed annually. In the US, the most common indications for a cholecystectomy are benign disease including acute cholecystitis, symptomatic cholelithiasis, and biliary dyskinesia. Regardless of the reason for the cholecystectomy the recommendation supported by literature is to send the gallbladder for histopathologic analysis [6]. While most histopathologic analysis of cholecystectomy specimens demonstrate benign findings, it occasionally provides the first evidence of malignancy in a patient. Most malignant pathology is subclinical primary biliary carcinoma [7]. Less commonly, the gallbladder can be a site for metastasis: melanoma is the most common primary tumor and has historically been estimated to be the source of 50–67 % gallbladder metastases [8,9]. A study published in 2009 by Yoon et al. reviewed 417 cases of malignant gallbladder disease and found that of these cases, 20 were due to metastatic disease. In this review the most common metastasis to the gallbladder was gastric cancer with other sites including colorectal cancer, hepatocellular carcinoma, renal cell carcinoma, melanoma, extrahepatic bile duct adenocarcinoma, uterine cancer, and mucinous adenocarcinoma of the appendix [10].

In our literature review, synchronous breast cancer metastatic to the gallbladder was found to be very rare. Of the reported cases of breast cancer metastasis to the gallbladder, lobular carcinoma was the most common primary tumor [3,7,11,12]. There are several case reports of metastatic breast cancer to the gallbladder found incidentally on elective cholecystectomy for biliary symptoms, however, most of these cases had a known history of breast cancer [7]. Our presented case is unique as the patient has no history of breast cancer. This case is more interesting in that the patient was subsequently diagnosed with two primary breast tumors, invasive ductal carcinoma and invasive lobular carcinoma, with only the lobular tumor being metastatic to the gallbladder and multiple osseous structures. This case supports the practice of sending all gallbladder specimens for histopathologic analysis [6]. Although malignant findings are rare, they are sometimes the only evidence of more insidious pathology for patients.

## Declaration of Competing Interest

The authors report no declarations of interest.

## Funding

Beaumont Farmington Hills, Department of Medical Education, paid for this report to be published.

## Ethical approval

Ethical approval not required for case report with no patient identifiers as there is minimal risk to patient.

## Consent

Written consent obtained from the patient by Richard Zekman, DO.

## Author contribution

Alona Salita – Wrote the paper.

Kita Mack – Edited the paper.

Marcos Rosado – Contributor and editor of paper.

John Pui – Provided pathology slides with descriptions.

Richard Zekman – Reviewed paper, provided Oncology perspective and treatment.

Kelly Dinnan – Reviewed paper, primary attending on case.

## Registration of research studies

Not applicable.

## Guarantor

Alona Salita.

Kita Mack.

Marcos Rosado.

Kelly Dinnan.

Richard Zekman.

John Pui.

## Provenance and peer review

Not commissioned, externally peer-reviewed.
